# How and Why Do Students Use Learning Strategies? A Mixed Methods Study on Learning Strategies and Desirable Difficulties With Effective Strategy Users

**DOI:** 10.3389/fpsyg.2018.02501

**Published:** 2018-12-14

**Authors:** Sanne F. E. Rovers, Renée E. Stalmeijer, Jeroen J. G. van Merriënboer, Hans H. C. M. Savelberg, Anique B. H. de Bruin

**Affiliations:** School of Health Professions Education, Maastricht University, Maastricht, Netherlands

**Keywords:** problem–based learning, desirable difficulties, self-regulated learning, learning strategies, mixed methods &lt, research methodology, grounded theory analysis

## Abstract

In order to ensure long-term retention of information students must move from relying on surface-level approaches that are seemingly effective in the short-term to “building in” so called “desirable difficulties,” with the aim of achieving understanding and long-term retention of the subject matter. But how can this level of self-regulation be achieved by students when learning? Traditionally, research on learning strategy use is performed using self-report questionnaires. As this method is accompanied by several drawbacks, we chose a qualitative, in-depth approach to inquire about students' strategies and to investigate how students successfully self-regulate their learning. In order to paint a picture of effective learning strategy use, focus groups were organized in which previously identified, effectively self-regulating students (*N* = 26) were asked to explain how they approach their learning. Using a constructivist grounded theory methodology, a model was constructed describing how effective strategy users manage their learning. In this model, students are driven by a personal learning goal, adopting a predominantly qualitative, or quantitative approach to learning. While learning, students are continually engaged in active processing and self-monitoring. This process is guided by a constant balancing between adhering to established study habits, while maintaining a sufficient degree of flexibility to adapt to changes in the learning environment, assessment demands, and time limitations. Indeed, students reported using several strategies, some of which are traditionally regarded as “ineffective” (highlighting, rereading etc.). However, they used them in a way that fit their learning situation. Implications are discussed for the incorporation of desirable difficulties in higher education.

## Introduction

Self-regulated learning (SRL) refers to the “process whereby students activate and sustain cognitions, behaviors and affects, which are systematically oriented toward the attainment of their goals” [(Schunk and Zimmerman, 1994), p. 309]. With the enormous increase in available information since the emergence of the Internet (Arbesman, 2013), SRL is becoming increasingly important in modern education. This can be especially daunting for students in a problem-based curriculum, as this approach places high demands on students' independent self-study and individual search for information (e.g., Kirschner et al., 2006). Students will need effective self-regulatory strategies in order to successfully navigate this educational landscape. As students often rely on ineffective, surface-level study strategies (Kornell and Bjork, 2007), it is important to understand what constitutes effective strategy use in a problem-based curriculum, and how to improve SRL in students not skilled in self-regulation.

An important concept in this regard is that of “desirable difficulties.” What constitutes as “desirable” when introducing difficulties into the learning process, at least from the students' perspective, will likely depend on the goals they set for learning. Learning goals can include long-term understanding and transfer, or simply a desire to pass an exam. When the aim is simply to pass the test, different learning strategies apply than when the focus is on long-term understanding and transfer. In fact, strategies which have a positive effect on long-term understanding and transfer, may even have a negative effect on learning in the short term and vice versa (Van Merriënboer et al., 1997; Helsdingen et al., 2011; Van Merriënboer and Kirschner, 2018). However, this short-term achievement will not prepare students for long-term, professional practice (Boud and Falchikov, 2006). From an educational perspective, the focus should therefore be on long-term retention and transfer. Indeed, as defined by Bjork (1994), creating desirable difficulties when learning refers to the process in which students use effortful learning strategies, with the aim of achieving long-term learning benefits, rather than surface-level strategies which are only effective in the short-term.

The traditional way of measuring students' strategy use is through self-report surveys (Panadero et al., 2016). These studies often reveal that students rely on ineffective strategies when studying. For example, Blasiman et al. (2017) found that over the course of a semester, students often relied on ineffective strategies such as reading notes and rereading text. Similarly, Karpicke et al. (2009) found that while students often rely on rereading strategies, few students use more effective strategies like retrieval practice. One of the drawbacks of this form of measurement is that students are usually confronted with a set of predefined strategy categories to choose from. Authors have raised questions about whether self-report questionnaires are able to gauge students' use of different learning strategies across different contexts and tasks (Winne and Hadwin, 1998; Perry and Winne, 2006; Schellings, 2011; McCardle and Hadwin, 2015), students' ability to recover the required information from their memory (Perry and Winne, 2006), the possibility of socially desirable answers (Bråten and Samuelstuen, 2007), and a potential tendency for students to rate the value they attach to a certain strategy rather than their actual use (Bråten and Samuelstuen, 2007; Bernacki et al., 2012). Another possibility is that students use certain strategies to regulate their learning which they do not recognize as belonging to a particular category (Veenman, 2011). Furthermore, it is possible that strategies which are traditionally treated as ineffective by these self-report questionnaires are in fact adapted by students to fit their learning situation and goals in an effective way. These expectations were the basis for exploration in the current study.

In order to overcome these difficulties, a more qualitative, in-depth approach to inquiring about students' use of learning strategies can be worthwhile in order to investigate how students successfully self-regulate their learning. Specifically, this rich form of data collection allows for the description of different contexts and learning tasks, allowing students to distinguish between different learning strategies used in different situations and for different goals, as well as how they potentially use seemingly “ineffective” strategies to adapt to a learning situation or goal. A qualitative approach to inquiry enables students to give more elaborate explanations for as to how and why they use particular strategies, as well as potential variations with regard to varying circumstances. By carefully constructing the questions, it should also be possible to distinguish between the value students attach to different strategies vs. their actual use. Furthermore, students' rich descriptions of their approaches to learning allow the researcher to identify strategies that students would be unable to correctly label in a questionnaire.

As a qualitative approach to data collection, the focus group method can have several advantages over traditional interviews. When using focus groups, participants' interactions with each other can yield insights that would not be possible to obtain with individual interviews (Kitzinger, 1995). In addition to being able to complement each other, participants have the opportunity to respond to each other's answers, making it easier to identify differences between their views. These differences can further be used to clarify the reasons behind participants' views (Kitzinger, 1994). Finally, with regard to social desirability, research has also found that focus groups, when compared to individual interviews, can actually induce participants to take a more critical stance (Watts and Ebbutt, 1987; Kitzinger, 1995). What matters here is to create a safe atmosphere for participants in which to express their views (Kitzinger, 1995).

For this study, we chose to focus on effective self-regulators, rather than making a comparison between effective vs. less effective students. Rather than focusing on the factors that influence effective self-regulation and the incorporation of desirable difficulties, the aim of this study was to take a step back and come to a comprehensive picture of what this effectiveness actually looks like.

In summary, in order to acquire more in-depth insight into the variation of students' strategy use and the reasons behind it, these considerations led us to choose a focus group approach to study students' self-regulation and incorporation of desirable difficulties into their learning. We complemented the focus group approach with a traditional learning strategy survey (cf., Hartwig and Dunlosky, 2012) to compare and contrast results between approaches and analyze the value of each. The research questions guiding this study were: How do highly effective self-regulating students in a PBL higher-education curriculum approach their learning? How do they incorporate desirable difficulties into this process?

## Methods

### Context

This study took place in the context of the first and second year of the 6-year undergraduate medical program at Maastricht University. This university uses a problem-based learning (PBL) format, in which learning takes place starting from authentic, real-world cases (Schmidt, 1983). Students work on these problems in small tutorial groups, typically consisting of approximately 10–12 students. These tutorial sessions are moderated by a tutor, who is expected to act as a facilitator, rather than as a knowledge transmitter. To structure the PBL process, Maastricht University uses a seven-step model called the Seven-Jump (Moust et al., 2005), consisting of clarification of terms, problem definition, brainstorming about possible explanations to the problem, structuring and analysis of the identified explanations, identification of learning questions, self-study, and post-discussion aimed at integrating individual students' findings. The first five steps take place in one tutorial group session, after which students individually study the literature to answer the learning questions outside the tutorial group. A few days later, the tutorial group gets together again to discuss their findings in the post-discussion, after which the cycle repeats for a new problem. In this curriculum, the academic year is divided into six courses, ranging between four to 8 weeks, each focusing on a specific multidisciplinary topic. At the end of a course, students are tested with an exam focused on the contents of this course (mostly multiple-choice).

Given its emphasis on students' independent literature search and self-study, the PBL format provides a fruitful context for the study of students' use of learning strategies and incorporation of desirable difficulties. Specifically, as students are required to find their own literature and use it to independently answer their learning questions they will need a range of strategies to manage this process and monitor their understanding, leading to a large pool of potential strategies for students to report on. This situation offers a unique potential to gain insight into what constitutes an effective approach.

### Participants

In order to come to a picture of effective strategy use and the incorporation of desirable difficulties for students in a PBL curriculum, we used a purposive sampling strategy (Ritchie et al., 2013). At the end of the first year (academic year 2013–2014), mentors of first-year undergraduate medical students were asked to identify students who they perceived to use effective learning strategies (the instructions for the mentors can be found in Appendix A in the Supplementary Material). Sixteen mentors identified 42 students for the study. These students were approached by e-mail to invite them for our study, to be held at the beginning of their second year (academic year 2014–2015). Thirty students (71%) indicated willingness to participate. Two students indicated it would not be possible to be present at the times the focus groups were held. Two students filled out the learning strategy questionnaire (see below) but did not attend the focus groups, and were therefore excluded from further analysis. The final number of students participating in the focus groups was therefore *N* = 26, of which 20 students were female, ages ranging between 18 and 23 years old (one student did not provide an age). The total number of students enrolled for the tutorial groups at the beginning of Year 2 was 298. Written informed consent was obtained from all participants prior to the start of the study. The study was approved by the ethical review board of the Netherlands Association for Medical Education (file number 402). Students were offered a small monetary gift voucher as a reward for their participation in the study.

### Learning Strategy Questionnaire

At least one week prior to the focus groups, students were asked to fill out a learning strategy questionnaire. We adapted the questionnaire used by Hartwig and Dunlosky (2012) to fit our PBL learning situation. Specifically, we adapted the wording of the questionnaire to refer to the tutorial group meetings that students encounter in the PBL setting. Furthermore, rather than asking students whether they do or do not use a specific strategy regularly (using a binary yes/no format), we used a Likert scale asking students how often they use these strategies while studying, ranging from 1 (never) to 5 (every study session). This was also applied to the question of whether students go back to course material after a course has ended, and whether students read study sources more than once. For the questions asking students on what parts of the day they study most and on what parts of the day they study most effectively, “evening” and “late night” were combined into one category (“evening”). Furthermore, the strategy questions were adapted to reflect the ones most relevant for the current educational context.

We dropped the question asking students whether they study more for open questions or multiple-choice questions, as the tests that medical students encounter in the program are mostly multiple-choice. The question of how students decide what to study next was posed as an open question. Finally, in order to reflect the focus of our study, we added four questions: (1) How did you develop the study strategies you are using now (open question, replacing the question if whether students' study strategies were taught to them by a teacher), (2) If you had the time and somebody would explain it to you, would you want to change your study strategies (yes/no), (3) What and why would you then want to change (open question), and (4) What kind of education would you most appreciate to change your study strategies? Think about: lectures, videos, practice with a trainer, etc. (open question). Finally, we added a question asking students for any further comments they may have. All questions not rated on a Likert scale (open questions and study times) were thematically coded by two raters. Inconsistencies were discussed until consensus was reached.

With this questionnaire we attempted to obtain a baseline measure of students' strategy use (*what* are the strategies that are used), to later complement this with the in-depth focus groups (*how* are the strategies used). In summary, the adapted questionnaire consisted of 10 questions assessing students' strategy use, using a Likert scale ranging from 1 (never) to 5 (every study session), as well as one question allowing students to list other strategies they use during studying. Furthermore, there were 12 questions inquiring about additional aspects of students' study behavior, for example, preferred study time (with five questions being open ended). Appendix B in the Supplementary Material provides an overview of the questionnaire.

### Focus Groups

Students were divided into four separate focus groups. Each focus group lasted ~1 to 1.5 h. Each focus group was moderated by the second author and observed by the last author and a student assistant. The second author is an educational scientist by background and specializes in qualitative methodology. The last author specializes in effective study strategies. She served as an observer, in order to avoid influencing the results or “leading” the participants. The student assistant observed as well and organized the focus groups. Based on a vignette approach, students were asked how they prepare for different educational activities in the PBL medical curriculum. A total of six vignettes was used (see Appendix C in the Supplementary Material for the interview protocol, including the vignettes used). These vignettes concerned the post-discussion, exam, progress test, skills lab, Pscribe (written assignments assessing students' pharmacotherapeutic reasoning) and extracurricular activities. To answer our research question related to students' learning strategies during self-study, we focused our analysis on the first two vignettes (post-discussion and exam). After 4 months, students were invited back for a second focus group meeting, in which we discussed preliminary results, in order to check our interpretation of the findings (member checking), and to see whether students were consistent in their reports. Two students did not attend the second meeting because the interview dates did not fit their schedule.

The interview protocol used for the focus groups can be found in Appendix C in the Supplementary Material.

### Analysis

All focus groups were audio recorded and transcribed verbatim. A constructivist grounded theory methodology (Charmaz, 2014) was taken when analyzing the data. In grounded theory, the aim is to generate a theory or understanding of a certain process (Creswell, 2007). In a process of iterative data analysis, the researchers go through the different steps of open coding (generating initial codes for data categories), axial coding (identifying a core phenomenon and its surrounding categories), and selective coding (connecting categories and developing the theory). We chose this approach due to our focus on understanding the *process* of effective strategy use and incorporation of desirable difficulties, with a strong interest in the conditions that support or hinder this process (Creswell, 2007).

Initial, open coding was done by the first author. This was done in a line-by-line fashion, in which representative codes were assigned to the participants' utterances. During this process, several meetings were held with the second and last author to discuss the codes. After arriving at an initial codebook, codes were related to each other in a process of axial coding. During this process, codes were compared and contrasted with each other, looking for connections in order to create themes from overlapping codes. This step was initially done by the first author, with the second and last author each coding a non-overlapping 25% of the codebook to ensure rigor. Findings from this step were discussed until consensus was reached. Results from the analysis were discussed with the third and fourth author. Finally, in a process of selective coding by the first, second and last author, themes were related to each other in order to come to an overarching model of the data.

## Findings

### Learning Strategy Questionnaire

Tables 1,2 show the results from the survey on students' strategy use and the additional aspects of students' study behavior, respectively.

**Table 1:** Means and standard deviations for students' responses on the learning strategy questions, from highest to lowest mean.

**Strategy**	***N***	**Mean**	**Standard deviation**
Self-testing	26	4.0	1.1
Summarizing	26	3.9	1.2
Mental imagery	26	3.9	0.8
Underlining/marking	25	3.5	1.3
Questioning	26	3.4	0.9
Self-explanation	26	3.3	1.1
Rereading	26	2.9	1.0
Co-studying	26	2.6	1.1
Cramming	26	2.2	1.1
Asking someone to test me	26	1.8	1.0

**Table 2:** Summary of students' responses to questions about additional aspects of their study behavior.

**Question**	***N***	**Answers**	**Number of responses**	**Mean**	**Standard deviation**
How do you decide what to study next?^a^ [open question]	26 (40 answers)	I study everything	1 (2.5%)	
		I study in a random order	1 (2.5%)	
		I use the course's structure	9 (22.5%)	
		Making a schedule ahead of time	11 (27.5%)	
		Using the order in which information is presented in sources	1 (2.5%)	
		Whatever costs least time	1 (2.5%)	
		Whatever I feel that I don't (fully) understand/find difficult	6 (15.0%)	
		Whatever I find interesting	1 (2.5%)	
		Whatever is due soonest	5 (12.5%)	
		Whatever is most important to me	3 (7.5%)	
		Whatever takes most work	1 (2.5%)	
Do you usually return to study material from an earlier course after a course has ended? [Please indicate on a scale from 1 (never) – 2 – 3 – 4 – 5 (always)]	26			2.7	0.8
When you study, do you usually read the book/article/other source more than once? [Please indicate on a scale from 1 (never) – 2 – 3 – 4 – 5 (always)]	26			2.9	1.0
Imagine that in the course of studying, you notice that you understand a certain concept in the text. What do you do?	24	Don't study it again	9 (37.5%)	
		Study it again later	15 (62.5%)	
What time of the day do you mostly do your studying?^a^	26 (39 answers)	Morning	14 (35.9%)	
		Afternoon	19 (48.7%)	
		Evening	3 (7.7%) 3 (7.7%)	
		No preference		
During what time of the day do you believe your studying is most effective?^a^	26 (34 answers)	Morning	17 (50.0%)	
		Afternoon	14 (41.1%)	
		Evening	1 (2.9%)	
		No preference	2 (5.9%)	
				
What do you usually do: Prepare for a tutorial group in one study session right before the tutorial group OR space out tutorial group preparation over multiple study sessions?	26	One study session	4 (15.4%)	
		Multiple study sessions	22 (84.6%)	
How did you develop the study strategies you are using now?^a^ [open question]	26 (33 answers)	Adjusting to requirements	5 (15.5%	
		Comparing with other students	2 (6.1%)	
		Experience	15 (45.5%)	
		Experimenting/trial and error	10 (30.3%)	
		Tips from staff	1 (3.0%)	
If you had the time and somebody would explain it to you, would you want to change your study strategies? [yes/no]	26	Yes	13 (50.0%)	
		No	11 (42.3%)	
		Maybe	2 (7.7%)	
What and why would you then want to change?^a^ [open question]	17 (20 answers)	Effective studying/efficiency	8 (40.0%)	
		Focus during lectures	1 (5.0%)	
		Improve study order	1 (5.0%)	
		Integrating/applying knowledge	2 (10.0%)	
		Making better summaries	1 (5.0%)	
		Making studying more fun	1 (5.0%)	
		Planning	4 (20.0%)	
		Start studying with other students	1 (5.0%)	
		Test taking strategies	1 (5.0%)	
What kind of education would you most appreciate to change your study strategies? Think about: lectures, videos, practice with a trainer, etc.^a^ [open question]	22 (29 answers)	Creating a mindmap or something visual	1 (3.4%)	
		Exercises	2 (6.9%)	
		Exercises with trainer	12 (41.4%)	
		Lecture followed by exercises with trainer	1 (3.4%)	
		Lectures	4 (13.8%)	
		More opportunities to ask questions	1 (3.4%)	
		Talking with fellow students about the learning materials	1 (3.4%)	
		Trying and discussing ideas of others (possibly of a trainer)	1 (3.4%)	
		Videos	5 (17.2%)	
		Written explanation with discussion session led by trainer	1 (3.4%)	

a *Students could provide multiple answers in response to these questions. Percentages therefore reflect proportions of the total number of answers given, rather than the total number of students*.

Interestingly, the students in our sample indicate a frequent use of the strategies regarded in the literature as effective, such as self-testing, questioning and self-explanation (Hartwig and Dunlosky, 2012; Dunlosky et al., 2013), indicating that our purposeful sampling strategy was effective. Furthermore, as indicated in Table 2, students report spacing their tutorial preparations over multiple sessions, indicating use of distributed practice (Dunlosky et al., 2013). However, as indicated by Table 2, students also report using some of the strategies that are typically viewed as ineffective for reaching long-term retention and transfer, particularly summarizing, mental imagery and underlining/marking (Dunlosky et al., 2013).

When responding to the question about which other strategies they use, strategies students reported (restricted to the ones not covered by the questionnaire) were: preparing their case on their laptop and shortly summarizing it before the tutorial group, writing out practical activities and going over this information during the exam week, drawing or writing out difficult things, making practice tests and correcting incorrectly answered items, watching videos, making diagrams after studying a case to summarize as much as possible, making concrete and compact cases, working in a disciplined manner, creating mind maps and drawings, drawing figures or pictures, rereading summaries, writing down and rereading difficult parts, printing out all cases and information from practicals and putting them together in one-folder to create an overview of the entire course, rehearsing lectures, and attentively working out learning materials in the case.

When responding to the question asking students whether they had any further comments, students emphasized the importance of lectures, active processing of learning materials through the creation of summaries, the added value of PBL and discussions during tutorial groups, and the importance of keeping order in the learning materials to avoid missing information.

In the focus groups, students were asked about their study approaches, in order to gain more insight into the ways in which they use their learning strategies.

### Focus Groups

**Figure 1:**
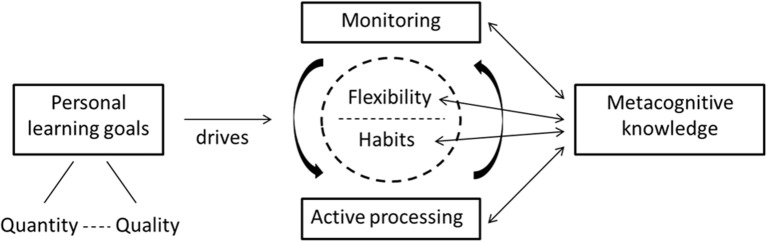
Model describing highly effective strategy users' approach to learning.

Using the constructivist grounded theory methodology, a model was constructed describing how highly effective strategy users approach their learning. The results of this process are depicted in Figure 1. In this model, students are driven by a personal learning goal, adopting either a qualitative or quantitative approach to learning. When learning, these highly effective strategy users are continually engaged in active processing of subject matter, while monitoring their understanding of the content and adjusting their approach when necessary. This process is guided by a constant balance between adhering to established study habits, while maintaining a sufficient degree of flexibility to adapt to changes in the learning environment, assessment demands and time limitations. Although students demonstrated metacognitive knowledge of the effectiveness of their strategies and the reasons for using them, this was not the case for all aspects of their strategy use. Indeed, students reported using several strategies which are traditionally regarded as “ineffective” (highlighting, rereading etc.), but used them in a way that helped them adjust to their learning situation and goal.

In the following, we will describe the different components of this model, the implications for students' self-regulation, and the incorporation of desirable difficulties into their learning.

#### Quantity and Quality

During the focus groups, many of the students described being driven by a personal learning goal, adopting a quantitative or qualitative approach to learning. Specifically, quantitatively oriented students used numerical indicators as the basis for their learning. For example, when referring to collecting information for the post-discussion, one student stated:

“*And then, yeah, just translate it a little bit and write it down in my own words. And uh, yeah, then I just have about fifteen pages usually. And when I have three pages I really feel like I, yeah, have too little*.”

Focus group 1, session 1, participant A

On the other hand, students adopting a qualitative approach emphasized the quality of their materials and of their understanding. Rather than focusing on how *much* material they had produced, these students would focus on how *well* they understood and remembered what they had studied. As one student explained:

“*Well, if you have 7 pages and you don't understand any of it, you haven't achieved anything in the end. You'll have a lot of material to study and when you study you can brag about having a 50-page case*.”

Focus group 1, session 1, participant B

#### Active Processing and Monitoring

During focus group discussions it became clear that students were continually engaged in active processing of the subject matter, while monitoring their understanding of the content and adjusting their approach when necessary. In this sense, students are incorporating desirable difficulties into their learning, as they are not content with passively reading the subject matter, but try to find ways to be actively engaged.

“*You should never literally copy an entire text. Or* [you should do it] *in the way he* [other participant] *does it, explain it or write it in your own words, but do something that makes it your own.”*

Focus group 4, session 1, participant A

In some cases, the PBL system at Maastricht University was indicated as a contributing factor to this active approach, as students are required to be able to discuss their findings in the post-discussions. This became clear in the words used to describe it:

“*I think that is really the key, treat the subject matter in an active way. You're in Maastricht, this is what they ask from you and it also just works.”*

Focus group 1, session 1, participant C

“*Well I had, yes in* [a different city] *I really had to learn from books. (…), so I think that that is just, that's not possible here, in Maastricht you also have to be able to tell everything coherently. So then I made a mix from that, that I, because I was good at studying from books, but that I could also reproduce it in the tutorial group.”*

Focus group 2, session 1, participant A

In addition to this active processing, students reported a continuous monitoring of understanding, and adjusting their learning when necessary. In many cases, this monitoring was achieved by various forms of self-testing. A commonly reported tactic for this was explaining the subject matter to another person, either physically or hypothetically:

“*(…) sometimes it is nice when people are like asking questions. Then I hear myself explaining it and then I hear whether I understand it, so to speak.”*

Focus group 3, session 1, participant A

“*And, uhm, when I look through my case at the end I should actually be able to explain each component that I discuss to someone else. I don't actually do that, but I should be able to.”*

Focus group 1, session 1, participant B

Also, students often used externally provided resources such as practice tests to test their knowledge and understanding. Interestingly, the strategies students reported using to correct learning when this monitoring revealed knowledge deficits, were mostly surface-level strategies such as rereading. However, self-testing is an important strategy to improve learning (Roediger and Karpicke, 2006) and has to be actively built into the learning process. The fact that students reported using practice tests and testing themselves indicated again students' willingness to incorporate desirable difficulties into their learning.

#### Habits and Flexibility

Students' learning process, as guided by their learning goal and characterized by active processing of subject matter and continuous monitoring of understanding, is further guided by a constant balance between adhering to established study habits, while maintaining a sufficient degree of flexibility to adapt to changes in the learning environment, assessment demands and time limitations. For example, students often indicated they had fixed times or places for studying, or a fixed order in which to process the materials for studying. At the same time, students also found themselves in situations where they had to adapt to changes in their learning situation and reported several strategies to maintain this flexibility. For example, one student indicated photocopying book sections in advance to be able to study when going home to parents during the weekends, thereby maintaining flexibility in time and location on which to study. This flexibility was also evident in students' strategy use. Students reported that they had experimented with strategies over the years, finding out “what works for them.” While some students indicated that they had not experienced the need to change their strategies, because they felt comfortable with their strategies and were happy with the results they produced, others indicated that they had used criteria such as their performance as benchmarks for whether or not they should adjust their strategies. As one student indicated:

“*I think this is something in which you are supposed to grow and if you keep telling yourself that your own strategy works and you score 6's* [points out of 10, 10 being the highest] *then you're actually doing something wrong. But then you're just, I would almost say lazy, you just don't feel like changing it*.”

Focus group 1, session 1, participant B

Furthermore, several students indicated adapting their strategies according to the demands of the test. Although some students reported using the same studying methods regardless of the way of questioning on the test, others indicated adapting their strategies depending on whether they would have to answer multiple choice questions (focusing on retention and recognition) or open questions (studying more, with a stronger focus on understanding).

#### Metacognitive Knowledge

Although students demonstrated metacognitive knowledge of the effectiveness of their strategies and the reasons for using them, this was not the case for all aspects of their strategy use. Students indicated in the second session that, when given a list of all strategies mentioned in the first session and asked to indicate which strategies they used most often, it was difficult to label these strategies properly. As one student indicated:

“*(…) with me mostly with visualizing, that I didn't realize that I was doing it or how I was doing it, until I wrote down that I was doing it. Then I thought, oh yes, I do this quite a lot.”*

Focus group 1, session 2, participant B

It was especially difficult for students to indicate how they monitored their understanding, or how they distinguished between important and less important topics and how deep to process the information. Many students indicated this was a “feeling,” or something they had learned from experience.

Furthermore, students reported using several strategies which are traditionally regarded as “ineffective,” such as highlighting and rereading of text (Dunlosky et al., 2013). However, students used them in a way that helped them adjust to their learning situation, by using the strategies in an active way. Although there were some exceptions (e.g., highlighting text in order to reread it afterwards), examples include repeating subject matter using different sources and media, making handwritten summaries to be actively engaged with the subject matter, paraphrasing in order to monitor understanding, or rereading text to check whether it still makes sense in the context of clinical practice.

In fact, in one of the focus groups students indicated the need to incorporate desirable difficulties into their learning process, emphasizing the wish to attain long-term retention, rather than short-term storage, in order to become a competent doctor after graduation. Students often recognized the need to invest effort in learning, as opposed to relying on low-effort surface-level strategies (for example, purposefully using English rather than Dutch books, as the additional effort required prevents a shallow reading of the text). An overarching theme in this regard was a focus on creating understanding, finding the logic in the educational material and making connections between different topics and educational activities, as opposed to for example rote learning or memorizing symptoms. One student explained:

“*I always do that* [check if you can apply the case to medical practice]*, I always try to make the case explainable. Just because I like that, then I know that I understand and when it is written down on sheets everywhere then I think oh, why is this value high or that value low. Or, because that lab test, oh yes, that makes sense too. It is not that I will think about what it is* [come up with a diagnosis], *but I do check to see if it makes sense or not”*

Focus group 2, session 1, participant B

In summary, the participants in our study use a variety of strategies to regulate their learning and to incorporate desirable difficulties into this process. In addition to active processing of subject matter and a continuous monitoring of understanding, participants understand the need to obtain long-term storage and understanding, rather than short-term results, in some cases prompted by the perspective of having to become a capable doctor.

## Discussion

This paper outlines the results of a study investigating highly effective strategy users' approaches to learning. As a starting point, a survey was administered to students asking about how their study strategies and how they approach their learning. Results from this survey indicated students' adherence to some highly effective strategies (e.g., self-testing), but also the use of some of the less effective strategies (e.g., highlighting). Afterwards, focus groups were organized in order to gain insight into how students use these learning strategies. Specifically, as survey data can provide insight into which strategies students use and how often they use them, the qualitative approach can clarify why students use these strategies, under which circumstances, and how flexible they are regarding their use.

Based on the focus groups, a model was constructed which describes how these students prepare for different learning activities. The first element in our model, as emanating from the focus groups was the distinction between quantitatively vs. qualitatively oriented students. The students who mentioned having a learning goal, expressed this in a way that suggests a sharp distinction between these two opposites: students are *either* quantitatively *or* qualitatively oriented. However, from a motivational or self-regulatory perspective, one would expect this variable to fall along a continuum (Ryan and Deci, 2000), with students leaning more toward either side of the spectrum depending on varying contexts and conditions. For example, it is conceivable that students who have a predominantly qualitative orientation might become more quantitatively oriented in the face of insecurities or time constraints. Conversely, generally quantitatively oriented students might adopt a more qualitative orientation when studying topics they are highly interested in. Possibly, students who did not express a learning goal might fall somewhere along this spectrum (a point we have tried to emphasize by adding the dotted line connecting the two opposites). Validating the polarized vs. continuous nature of this distinction, as well as determining the factors that influence students' respective orientations, could be an interesting avenue for future research.

The second theme concerned students maintaining a continuous balance between established habits vs. a flexibility to meet changing demands. Indeed, this would make sense from a desirable difficulties perspective, as these students do not “give up” in the face of changing demands, but rather persist and adapt to the situation in order to reach their goals. Earlier research also correlated flexibility (termed adaptive control) with self-regulated learning, deep processing, and a propensity to undertake effortful cognitive activities (Evans et al., 2003). In terms of implications, several follow-up questions can be asked. First, what is the optimal combination between habits and flexibility? Will this balance be different in less effective students? What are students' core habits? What should be flexible, and what should be stable? What can be taught? Interventions should focus on optimizing this balance. Monitoring of understanding could be at the core of these interventions. When students have an accurate insight into which aspects they do and do not understand, and which strategies lead to a better understanding, it can be easier to make decisions about which strategies need to remain stable, and which should be adapted.

The third theme arising from the data, which characterized students' learning process, was students' continuous engagement in active processing of the learning material and monitoring of understanding. In addition to being aspects of effective (self-regulated) strategy use (Zimmerman, 1990; Dunlosky et al., 2013), it is also possible that this result can (at least partly) be attributed to the PBL curriculum in which this study took place, as these learning methods are hallmarks of this instructional approach (Hmelo-Silver, 2004; Loyens et al., 2008). Indeed, one of the students in the focus group even indicated the problem-based curriculum as a reason for adopting an active approach to learning. Given the fact that this study has only been carried out in a PBL context, it is difficult to disentangle these influences. Future studies could seek to unravel these factors further.

The final theme emerging from the focus groups concerned students' metacognitive knowledge. Interestingly, students reported using several strategies which traditional self-report questionnaires tend to treat as “ineffective,” but used them in an active way to help cope with the demands of their specific learning situation. This indicates that what matters most is not *which* strategies students use, but rather *how* they use them. In other words, students *adapted* strategies to fit their particular learning situation. Indeed, students' adaptability in their strategy use has been identified by other authors as an important feature of effective self-regulation in students (Broekkamp and Van Hout-Wolters, 2007). This sense of flexibility was also evident in other parts of the model, where students maintained a continuous balance between established study habits on the one hand, and a sense of flexibility to deal with changes on the other.

Another reason for students' use of surface-level learning strategies could be the form of assessment. Students are often assessed with multiple-choice question tests or open question tests focused solely on short-term retention of information. Several studies have found that students will adapt their strategies based on what they perceive will be expected of them on the examination (Thomas and Rohwer, 1986; Broekkamp and Van Hout-Wolters, 2007). Indeed, students in our study indicated changing their strategies according to whether questions would be asked in a multiple choice vs. an open question format. In this sense, rather than being “ineffective,” these surface-level strategies could be interpreted as being highly efficient in terms of the (short-term) goal students are aiming to achieve, if this goal is to obtain a good grade on the retention-based exam (Morris et al., 1977). If the goal of the curriculum is for students to strive for deep-level processing and understanding, the test demands need to be aligned with this objective (Broekkamp and Van Hout-Wolters, 2007), asking questions that will require this approach from students.

On the other hand, several students indicated an understanding of the need to obtain long-term retention and understanding, an inclination that seemed to be promoted by a desire to become a capable doctor. This can have important implications for interventions aimed at improving self-regulation for students who are less skilled self-regulators. Specifically, if interventions would focus on aiding students in attaining a clear perspective of their goals and long-term profession, this could improve their self-regulatory behavior and intention to build in desirable difficulties into their learning. Although we did not originally set out to investigate the link between students' learning behavior and their future time perspective, previous work has been done to establish this link, with research indicating that students' long-term time perspectives are associated with adaptive self-regulatory strategies and deep cognitive processing (Bembenutty and Karabenick, 2004; de Bilde et al., 2011). As these studies are mostly correlational, the direction of these effects is not entirely clear. Future research could try to establish the direction of causality by employing a longitudinal (de Bilde et al., 2011) or experimental approach.

The model identified can elaborate on existing theoretical models of metacognition by explicating the criteria students use to monitor and control their learning and how they adapt their strategies to fit their learning needs. For example, Nelson and Narens (1990) outline a theoretical framework in which students' allocation of study time is determined by their judgments about the difficulty it takes them to master certain information (ease of learning; EOL), their judgments about how well they have mastered certain recallable information (judgments of learning; JOL), and the degree to which they believe they have previously known currently unrecalled information (feeling of knowing; FOK). Their research found that students will allocate extra study time based on their EOL, JOL, and FOK judgments, with students studying general information items generally allocating extra study time to information with a lower EOL (meaning they are judged to be harder), higher FOK, and lower JOL. Also when it comes to the allocation of *restudy*, students will allocate this restudy time to information they judge as poorly learned (Nelson et al., 1994). The current study adds to this literature by shedding light on some of the criteria students may use to make these judgments. Specifically, students seem to focus on qualitative or quantitative criteria for making these judgments. Furthermore, for FOK, Nelson and Narens (1990) indicate that these judgments monitor the recallable aspects of the information a student has in memory (such as whether they have used it to correctly answer a question before). This could potentially explain the differences between the qualitative and quantitative orientations found in our study. For some students, the qualitative aspects related to the studied information may be hard to recall. For example, some of the information may never have been tested yet, making it difficult for students to derive these judgments. This may lead them to focus on more easily recallable, quantitative information instead.

Following this line of reasoning, this focus on easily recallable, quantitative aspects of learning may lead students to adopt more surface-level strategies, as these might be sufficient to satisfy the quantitative criteria. Indeed, Koriat (1997) found that extrinsic cues are less informative for students' JOLs than intrinsic cues, and these inaccurate JOLs could in turn lead to inadequate study strategies. Although students in our study seemed to follow the same general path of self-regulation, the qualitative approach might lead to more elaborative learning strategies and incorporation of desirable difficulties. However, a focus on quantitative criteria is apparently sufficient for students to pass their exams and be successful in university (a point which was already elaborated upon above). However, we do not have any information about their long-term retention. Future studies should focus on more elaborative learning outcomes and longer retention intervals, to further unravel the potentially differential effects of the different approaches to learning.

This study has several limitations. First, our focus groups were limited to second-year undergraduate medical students who were effectively self-regulating their learning. Given the PBL context in which these students are learning, this provided a fruitful basis to start from when investigating effective students' approaches to learning, but we cannot be sure about how these findings relate to other student populations. Furthermore, our study was limited to students from the undergraduate medical program. It is possible that there are characteristics in this program, which are not easily transferable to programs focusing on other domains. A specific example of this can be found in the long-term perspective that several students indicated as the basis for their desire to understand the subject matter, as hinted at above. In a study program like Medicine, the end goal of becoming a doctor is quite clear. In many other undergraduate programs, this long-term perspective may be less evident. Future research could look into what constitutes effective self-regulation in other study programs and other, non-PBL oriented universities. Furthermore, although the purpose of this study was to illustrate effective self-regulation rather than to contrast different groups of students, it would be interesting to see what picture will emerge when asking the same questions to low self-regulating students. We have tried to ensure replicability by providing rich descriptions of context, methods, and results, in an attempt to increase opportunities for judgments of transferability.

Related to the distinction between effective vs. ineffective strategy users is the questions of whether we were able to correctly identify which students were effective strategy users. We used students' mentors as informants for our purposeful sampling strategy. We have confidence in this strategy, as mentors are among the few key persons who have a bird's eye view of students' overall performance, for both the entire duration of the program, as well as in comparison to other students. They also discuss students' learning strategies at least two times during the first year in an individual mentor meeting. However, their judgments are inherently subjective, and although they were given instructions on what is meant by effective strategy users, we have no insight into their decision making when they selected these students. Although it was a conscious decision not to include grades as a measure of self-regulation (as students using shallow strategies may very well obtain good test results in the short term), it could be worthwhile to think about other ways to triangulate students' strategy effectiveness.

Finally, we chose to use learning questionnaire used by Hartwig and Dunlosky (2012) as a starting point for our study, in order to build further on this work and demonstrate the added value of the focus groups in this context. However, as this survey measures each strategy by only one item, it was not possible to compute reliability or internal consistency estimates. This problem is mitigated by the fact that we used the survey as a starting point for our focus groups, rather than conducting analyses analyzing differences between groups or as a result of some intervention. However, the research design could be strengthened by adding more items per strategy, in order to be able to make inferences about the reliability and internal consistency of students' responses.

Overall, this study contributes to the literature by providing an in-depth, qualitative description of how highly self-regulated medical students in a PBL curriculum approach their learning and build in desirable difficulties in their learning process. This model can serve as a framework for further study into the various factors that influence (effective) self-regulation, and as a starting point for designing interventions focused on improving strategy use in less effective students.

## Author Contributions

AdB and RS were responsible for the design and data collection of the study. SR performed analysis of the data, in close collaboration with AdB and RS. SR drafted the article, incorporating edits, and feedback from all other authors (AdB, RS, JvM, and HS). All authors made a substantial contribution to the interpretation of the data for this work.

### Conflict of Interest Statement

The authors declare that the research was conducted in the absence of any commercial or financial relationships that could be construed as a potential conflict of interest.
