# Corrigendum: How and Why Do Students Use Learning Strategies? A Mixed Methods Study on Learning Strategies and Desirable Difficulties With Effective Strategy Users

**DOI:** 10.3389/fpsyg.2020.00261

**Published:** 2020-02-20

**Authors:** Sanne F. E. Rovers, Renée E. Stalmeijer, Jeroen J. G. van Merriënboer, Hans H. C. M. Savelberg, Anique B. H. de Bruin

**Affiliations:** School of Health Professions Education, Maastricht University, Maastricht, Netherlands

**Keywords:** problem–based learning, desirable difficulties, self-regulated learning, learning strategies, mixed methods&lt, research methodology, grounded theory analysis

In the original article, there was an error. The article states that “Results from the analysis were discussed with the second and fourth author. Finally, in a process of selective coding by the first, second and fourth author, themes were related to each other to come to an overarching model of the data” (p. 4).

However, as stated correctly in the Author Contributions, coding was conducted in collaboration between the first, second and *last* (i.e., fifth instead of fourth) author. Results from the analysis were discussed with the *third* and fourth author.

A correction has been made to the Methods section, subsection *Analysis, Paragraph 2*:

Initial, open coding was done by the first author. This was done in a line-by-line fashion, in which representative codes were assigned to the participants' utterances. During this process, several meetings were held with the second and last author to discuss the codes. After arriving at an initial codebook, codes were related to each other in a process of axial coding. During this process, codes were compared and contrasted with each other, looking for connections in order to create themes from overlapping codes. This step was initially done by the first author, with the second and last author each coding a non-overlapping 25% of the codebook to ensure rigor. Findings from this step were discussed until consensus was reached. Results from the analysis were discussed with the third and fourth author. Finally, in a process of selective coding by the first, second and last author, themes were related to each other in order to come to an overarching model of the data.

The authors apologize for this error and state that this does not change the scientific conclusions of the article in any way. The original article has been updated.

