# Supplementary material for: How and Why Do Students Use Learning Strategies? A Mixed Methods Study on Learning Strategies and Desirable Difficulties With Effective Strategy Users
Source: Front Psychol. 2018 Dec 14;9:2501. doi: 10.3389/fpsyg.2018.02501 (PMC6302009; doi:10.3389/fpsyg.2018.02501)
Supplement: Supplementary file 1 [file Data_Sheet_1.pdf]

Appendix A: Mentor instructions for identification of effective strategy users

Dear Medicine Year 1 mentors,

With permission of [the coordinator] I am e-mailing you with a question. Do you also notice that some students use very efficient study strategies, while others struggle with planning their study, how to make summaries and how to prepare for the exam? I am an educational researcher at the department of educational development & research, and I study metacognition and study strategies. I would like to map the effective study strategies of students, because I think other students can learn a lot from the ‘best practices’ of their fellow students and to see how these strategies correspond with ‘evidence-based’ strategies.

What do I want to ask from you?
For this study I would like to interview first-year Medicine students with good study strategies. In order to identify these students, mentors are the best suited. Throughout the entire previous year, they have witnessed the students from a close range. We are referring both to students who study well (have a good way of preparing for the tutorial group and exam), as well as students who are good at planning their study. **Would you be able to e-mail me the names of two or three students from your mentor group in this academic year who you think had the best study strategies?** At this point, this should be fresh in your memory. I will then contact the students to ask them if they would like to participate in the interview (this is of course voluntary, completely anonymous, and for a monetary reward). We plan to have the interviews in October 2014. This is the only way to reach the right students for the study, so I am looking forward to your responses (preferably as soon as possible, before July 11th at the latest).

Anique de Bruin.
