# Supplementary material for: How and Why Do Students Use Learning Strategies? A Mixed Methods Study on Learning Strategies and Desirable Difficulties With Effective Strategy Users
Source: Front Psychol. 2018 Dec 14;9:2501. doi: 10.3389/fpsyg.2018.02501 (PMC6302009; doi:10.3389/fpsyg.2018.02501)
Supplement: Supplementary file 2 [file Data_Sheet_2.pdf]

**Appendix B: Learning strategy questionnaire administered prior to the focus groups**

Please indicate how often you use these strategies (Please indicate on a scale from 1 [never] – 2 [almost never] – 3 [sometimes I do, sometimes I don't] – 4 [almost always] – 5 [every study session]

1. Rereading of books or articles
2. Making a summary
3. Underlining/marking text
4. Explaining to myself what I am reading
5. Trying to form a mental image (an image in my head) of what I am reading
6. Testing myself with practice tests or self-made test questions
7. Asking someone else to test me
8. Asking questions to other students (outside of the tutorial group)
9. 'Cramming' on the night before the test
10. Studying with friends/other students
11. Other (please indicate which ones)

Further questions

1. How do you decide what to study next? [open question]
2. Do you usually return to study material from an earlier course after a course has ended? [Please indicate on a scale from 1 (never) – 2 – 3 – 4 – 5 (always)]
3. When you study, do you usually read the book/article/other source more than once? [Please indicate on a scale from 1 (never) – 2 – 3 – 4 – 5 (always)]
4. Imagine that in the course of studying, you notice that you understand a certain concept in the text. What do you do? [Please indicate: Don't study it again OR Study it again later]
5. What time of the day do you mostly do your studying? [Please indicate: morning, afternoon, evening]
6. During what time of the day do you believe your studying is most effective? [Please indicate: morning, afternoon, evening]
7. What do you usually do: Prepare for a tutorial group in one study session right before the tutorial group OR space out tutorial group preparation over multiple study sessions?
8. How did you develop the study strategies you are using now? [open question]
9. If you had the time and somebody would explain it to you, would you want to change your study strategies? [yes/no]
10. What and why would you then want to change? [open question]
11. What kind of education would you most appreciate to change your study strategies? Think about: lectures, videos, practice with a trainer, etc. [open question]
12. Do you have any other comments? [open question]
