# Supplementary material for: How and Why Do Students Use Learning Strategies? A Mixed Methods Study on Learning Strategies and Desirable Difficulties With Effective Strategy Users
Source: Front Psychol. 2018 Dec 14;9:2501. doi: 10.3389/fpsyg.2018.02501 (PMC6302009; doi:10.3389/fpsyg.2018.02501)
Supplement: Supplementary file 3 [file Data_Sheet_3.pdf]

Appendix C: Interview protocol used for the focus groups

Interview guide second year students Medicine - Study strategies

Learning Strategies of Medical Students: Improving Learning Effectiveness

Meetings: 26 November, 10:00-12.00, 1 December: 16:00-18:00, 3 December: 11:00-13:00 & 16:00-18:00

Leading Research Question

How do undergraduate medical students prepare for educational meetings and exams, and how do they self-regulate their learning?

- To what extent do they use evidence-based strategies as self-testing and spacing? What are the different varieties of evidence-based strategies as self-testing and spacing that students use?
- What types of activities do they undertake to regulate their study behavior?
-

TO DO BEFORE THE START OF THE INTERVIEW

- **Informed consent**: To emphasize the research goal again, to declare that the participant is allowed to leave at every moment and that their results will be anonymized. They will receive a copy of the transcripts as a complement.
    - **NB Let them sign the form!**

INTERVIEW

Introduction

Research has shown that students in higher education rarely use effective study strategies. This leads to suboptimal learning results. Changing study strategies is often desired, but it is often not self-evident how this can be achieved and which alternatives are available.

In this research we want to interview students with effective study strategies to make an overview which strategies they use, to view how these correspond with scientifically proven strategies and which strategy variants they use. We also want to hear about their ideas and experiences about how they change their strategies and regulate their study behaviour.

Step 0
To give a global explanation of the goal of this research ("to get an overview on how students prepare for different study activities and which study strategies they use").

Step 1
To discuss different study strategies and the usage of them by students.

Procedure Step 1:

- Through strategies mentioned in the questionnaire (filled in by the students) the moderator will ask the students to describe in which extent they know and use these strategies.
- Students receive a sheet with different study strategies.
- After students have read this sheet, the next things will be discussed:
  - To which extent students use and know these strategies
  - To which extent the students use the strategies, depending on the assignment, education activity or other factors
  - To which extent students are aware of how they plan and regulate their study [n.b. to what extent students consciously plan and regulate their study (not planning in the sense of time management. If students tend to move in that direction, guide them in another direction)]

Step 2
Enhancing different study strategies and preparation on educational activities

Procedure Step 2:

- Through several vignettes about different educational activities the moderator will ask students to describe how they would prepare for these educational activities, which strategies they use for this and to name concrete examples.
- Students will get a sheet book with one vignette on each page.
- After students have read the vignette, the moderator will ask the questions listed below (questions with step 1} per vignette.
- The pile of vignettes will be worked through one by one.
- After discussing the vignettes, the moderator will ask for a summary:
  - Which strategies do you most often use and why?
  - How did these strategies develop?
  - What is the best tip for other students to improve their study strategies?

Questions according to Step 2 (per vignette):

1. APPROACH: how do you prepare for this educational activity?
2. EXAMPLE APPROACH: Can you give an example on how you prepared?
3. VARIATION IN STRATEGIES: to what extent do you vary in your approach and which other approaches do you use?
   [N.B. questions 1,2 and 3 keep asking about self-testing and spacing]
4. SELF-REGULATION: Do you verify if you have prepared enough? How do you do that and what do you to be better prepared? N.B. The use of 'self-testing' can be emphasized here.
5. FEEDBACK: How do you cope with the feedback you received on this educational activity? And with information outside of the regular materials (tutor instructions, summary, information from tutor e.g. learning goals)?
6. CONFIDENCE: Are you satisfied about your preparation?
7. CHANGE: If you would change something about your preparation, what would it be?

Vignettes
1. Case

As usual you will get a new case to prepare before the tutorial meeting next Monday. The subject is a little bit of repetition but most parts are totally new. How do you prepare your case? How do you look for literature and resources for your case and how do you estimate how much time you need to prepare? Could you explain how you would prepare for the tutorial meeting?

2. Skillslab (excluded from analysis)
The OSCE test is coming later in the year. You already heard from several people that the test is going to be hard and everything has to be according to the rules. How would you approach this OSCE test? How did you do that with previous feedback sessions? Do you make use of Teacher-Independent-training?

3. Exam
You are just starting your 8-week course and know that the course is going to be heavy and hard. There are no tests in between, so there is only one chance to pass this course. The cases are very different. A couple of cases are about the same subject while the other cases are about several different subjects. How do you prepare for an exam with only multiple choice questions? And how do you prepare for an exam with open questions? And what do you do when it is a combination of multiple choice and open questions?

4. Progress test (excluded from analysis)
In one and a half month the progress test is coming up. Last year it was easy but this year it is different, because you also have to remember the knowledge of the last courses. The knowledge you have to know keeps increasing. How do you prepare now for the VGT? How do you cope with feedback (PROF system)?

5. Pscribe (excluded from analysis)
In the beginning of the year you hear about a pharmacology assignment that has to be handed in in three weeks. You are allowed to work together if you wish, but it is not allowed to hand in the same assignment. You already know a little bit about the subject, but this knowledge needs to be refreshed. How do you approach this assignment?

6. Extra-curricular assignment (excluded from analysis)
In the beginning of the year you get to know that there is a surgical assignment due in 4 months. This assignment consists a report about a specialized subject. You cannot obtain information as easily as in usual in the commonly known books. Nobody you know has the same assignment. How do you approach this assignment?

Protocol 2nd round Focus groups Medicine year 2

Goal meeting: To obtain a rank of advisable study strategies per educational activity to recommend to students.

Different study strategies used by students in every educational activity in the first meeting are placed together. The question is to rank these study strategies from most to least advisable to other students.

Concrete:

1. Make, after discussion, a top 5 of most advisable study strategies (from most to least) per educational activity. [this might be not achievable for all educational activities. Do this for at least the tutorial, skillslab, exams and progress test].

2. Also declare why you recommend these 5 strategies and why this order.

Thank students for participating and distribute Iris cheques (vouchers), let students sign the form.
